# Impact of phthalate exposure on gestational diabetes mellitus: a systematic review

**DOI:** 10.3389/fendo.2025.1638655

**Published:** 2025-09-22

**Authors:** Tridip Mitra, A. H. Anil Kumar, Rithika Thangaraju, Shakthi Mani S. M., Sajeetha Kumari R., Rajiv Janardhanan

**Affiliations:** ^1^ Division of Medical Research, SRM Medical College Hospital and Research Centre, SRM Institute of Science and Technology, Chengalpattu, Tamil Nadu, India; ^2^ Department of Obstetrics and Gynecology, SRM Medical College Hospital and Research Centre, SRM Institute of Science and Technology, Chengalpattu, Tamil Nadu, India

**Keywords:** gestational diabetes mellitus, phthalates, urine, pregnancy outcomes, systematic review

## Abstract

**Background:**

Gestational Diabetes Mellitus (GDM) is a transient diabetogenic state that often leads to adverse maternal and fetal outcomes. The rising burden of exposure to endocrine-disrupting chemicals like phthalates essentially disrupts the tightly regulated endocrine system, thereby modulating the insulin signaling pathways, leading to GDM.

**Objective:**

In the present work, a systematic review was performed to examine the probable relation between maternal exposure to phthalates, as endocrine-disrupting compounds, and GDM.

**Methods:**

Relevant studies from their inception to April 2025 were identified by searching PubMed, Embase, Scopus, and Science Direct. The data were screened using the Rayyan tool, and the risk of bias was assessed using the New Castle Ottawa Scale selection tool.

**Results:**

We identified 13 studies that showed a significant presence of phthalates in the urine samples of GDM patients. 5 phthalate secondary metabolites, Monoethyl Phthalate, Monobutyl phthalate, Mono-Isobutyl Phthalate, and Monobenzyl Phthalate and the primary phthalate Di(2-ethylhexyl) Phthalate were found to be most commonly present in the urine samples of the GDM patients.

**Conclusion:**

Urinary phthalate levels can be used as a non-invasive biomarker for GDM, thereby also reducing the risk of associated adverse pregnancy outcomes.

**Systematic review registration:**

https://www.crd.york.ac.uk/prospero/, identifier CRD420251023656.

## 1 Introduction

Endocrine-disrupting chemicals (EDCs) are essentially exogenous compounds that are capable of interfering with the normal functioning of the hormonal system (1). They have been known to mimic, block, and disrupt endogenous hormonal signals, affecting homeostasis, development, reproduction, and metabolism. EDCs are omnipresent and are found in our everyday lives, present in cleaning products, industrial chemicals, and personal and home care products (2). Phthalate, which is a group of synthetic chemicals used as plasticizers in polyvinyl chloride, are one of the most widely studied classes of EDCs (3).

Phthalates are essentially synthetic diesters of phthalic acid. They are mainly used as plasticizers to increase the flexibility of polyvinyl chloride (PVC) and other polymers (4). Phthalates are primarily classified into high molecular weight (HMW) and low molecular weight (LMW) phthalates, with each having distinct chemical structures and applications (5). Common HMW phthalates include di(2-ethylhexyl) phthalate (DEHP), butyl benzyl phthalate (BBzP), and diisononyl phthalate (DINP), which are commonly found in medical devices, flooring, food packaging, and automotive products (6). LMW phthalates, such as diethyl phthalate (DEP), dimethyl phthalate (DMP), and dibutyl phthalate (DBP), are extensively used in daily personal care products, including perfumes, lotions, and cosmetics.

Human exposure to phthalates is pervasive and primarily occurs through inhalation, ingestion, and dermal absorption. Research has found that one of the most significant sources of exposure to phthalates is dietary intake through contaminated food and beverages stored in plastic containers or wrapped in plastic films (7, 8). Additionally, human exposure to phthalates via personal care and cosmetic products, which contain phthalates as solvents and fragrance stabilizers, is also a matter of great concern (9). High concentrations of phthalates in airborne particles and settled indoor dust, particularly in environments with high PVC content, are also known to pose potential harm to humans (10). Studies have also shown that even medical devices contain phthalate-based plasticizers, particularly those made with DEHP-containing tubing, such as IV bags and catheters (11, 12).

Research on phthalates has been gaining traction due to their potential role in disrupting reproductive and metabolic health, especially among vulnerable populations such as pregnant women. Phthalates can cross the placental barrier and disrupt maternal-fetal metabolic signaling, and the vulnerability of pregnant women to phthalate exposure is of special concern.

Gestational diabetes mellitus (GDM), defined as glucose intolerance first recognized during pregnancy, poses substantial health risks for both mother and fetus (13), including Hypertensive Disorders of Pregnancy (HDP), and future risk of type 2 diabetes in mothers. GDM can also lead to macrosomia, obesity, or metabolic dysfunction in the offspring (14). GDM can broadly be classified into GDMA1 and GDMA2 (15). GDMA1 is diet-controlled, meaning blood sugar is managed through nutrition and exercise alone, posing lower risks (15). GDMA2 requires medication (insulin or oral agents) due to uncontrolled glucose levels, increasing risks of fetal macrosomia, neonatal hypoglycemia, and maternal type 2 diabetes post-pregnancy (15). Early diagnosis and strict monitoring are crucial for both types to ensure healthy outcomes for mother and baby. The exposure pattern to these EDCs can be dietary, behavioral, or residential (16, 17). The most common risk factors for GDM include age, obesity, and family history. New evidence from contemporary studies emphasizes the role of environmental exposures, particularly EDCs such as phthalates, to be increasingly relevant contributors (17). It was found that around 50% of GDM patients appear to be prone to lifestyle stressors such as endocrine-disrupting chemicals (17, 18). Additionally, various studies have found that phthalate exposure can interfere with glucose and lipid metabolism, pancreatic β-cell function, and insulin sensitivity (19, 20). Studies have shown that phthalates have been shown to activate peroxisome proliferator-activated receptors (PPARs), which play a crucial role in lipid metabolism and adipogenesis, which in turn influences insulin resistance (21).

While extensive research has examined the relationship between phthalate exposure and GDM, there is a lack of systematic synthesis of findings across different geographic, socioeconomic, and clinical settings. Additionally, previous researchers have rarely considered contextual factors such as specific phthalate metabolites measured. Moreover, they have not considered the regional disparities in the prevalence of GDM and phthalate exposure (**Supplementary Table S1**). It is widely known that both GDM prevalence and phthalate exposure vary considerably across the globe, with considerable differences being observed in high-income countries (HICs), low and middle-income countries (LMICs), and low-income countries (LICs) (22).

Thus, this study aims to address these gaps by collating and analyzing the existing evidence on phthalate concentrations in women diagnosed with GDM across diverse populations, focusing specifically on biomarker-based studies. Additionally, this study seeks to assess whether phthalate exposure is consistently elevated in GDM cases compared to controls and to evaluate the magnitude and direction of associations across different contexts.

Furthermore, the study contributes to the broader field of environmental reproductive epidemiology by advancing our understanding of how ubiquitous environmental pollutants may intersect with maternal and fetal health outcomes. Thus, the main objective of this review is to systematically evaluate and examine the probable relation between maternal exposure to phthalates, as endocrine-disrupting compounds, and GDM. The findings of the study could be useful to provide evidence that can inform future research priorities, clinical guidelines, and environmental health policies aimed at safeguarding maternal metabolic health.

## 2 Methods

### 2.1 Registration

The systematic review was performed as outlined *a priori* in the registered protocols (PROSPERO registration ID CRD420251023656). Ethical approval was not required for the systematic review as these were secondary studies using published data.

### 2.2 Eligibility criteria

We included original reports that analyzed the phthalate levels in the urine samples of GDM patients (cases) and non-diabetic subjects (controls) in human trials. All case-control, cross-sectional and cohort studies were included in the study. Studies utilizing animal models and cell lines were excluded. The studies focused on the estimation of phthalates derived only from urine samples were included in our analysis. Study population, intervention, comparator, outcome, and study design (PICOS) parameters were predefined for objective and reproducible analysis.

### 2.3 Population

Any human study of GDM was included. Studies involving *in vitro*, ex vivo, or pre-clinical animal models of GDM were excluded.

### 2.4 Intervention

Studies included in our analysis did not deal with administering insulin dosages or any other interventions on human subjects.

### 2.5 Comparator

The main comparators were the association between urinary phthalate concentrations and GDM and related complications. Non-comparative studies were excluded.

### 2.6 Outcome

The main outcome of the study was the correlation of urinary phthalate concentrations with GDM. The secondary adverse pregnancy outcomes associated with GDM were also observed.

### 2.7 Study design

All English-language, full-text, clinical studies comparing urinary phthalate levels between pregnant women with and without GDM were included. Review articles, non-comparative studies, commentaries, editorials, case reports, case series, and other study types were excluded. Studies investigating the concentration of phthalates from urine samples in GDM patients were included. Studies dealing with other EDCs (heavy metals, parabens, bisphenols, triclosan, PFAS, organophosphates) were also excluded from the study. *In Vitro* studies, animal model studies or studies dealing with type 2 diabetes mellitus were also excluded from the study.

### 2.8 Search strategy

This study was performed based on the Preferred Reporting Items for Systematic Reviews and Meta-Analyses guideline (PRISMA 2020 statement) (http://www.prisma-statement.org/). A literature search strategy was developed according to the four different parameters of the study question (participants, intervention, comparison, and outcome) and the study design. PubMed (pubmed.ncbi.nlm.nih.gov), Scopus (www.scopus.com), Embase (www.embase.com), and Science Direct (www.sciencedirect.com) electronic databases were used to identify eligible original articles published up to April 5, 2025. The published articles in these electronic databases from last 10 years were included. The search terms that were used in these databases are “((Phthalate) OR (Phthalates) OR (Phthalate esters) OR (Phthalate metabolites)) AND ((Gestational Diabetes Mellitus) OR (Gestational Diabetes)) AND (Urine) AND (Human)”.

### 2.9 Data analysis

The data screening was done with the help of the Rayyan tool. The risk of bias was analyzed using the New Castle Ottawa Scale selection tool.

### 2.10 Comparison of phthalate concentration

The concentration of the phthalates that was most common among the included studies were compared using a heat map. All the values were converted to μg/L before comparison. The values of extreme variations in phthalate concentrations across studies were adjusted using a log 10 scale. This transformation ensures all values are visually interpretable, while still preserving relative differences.

## 3 Results

### 3.1 Study selection

**Figure 1:**
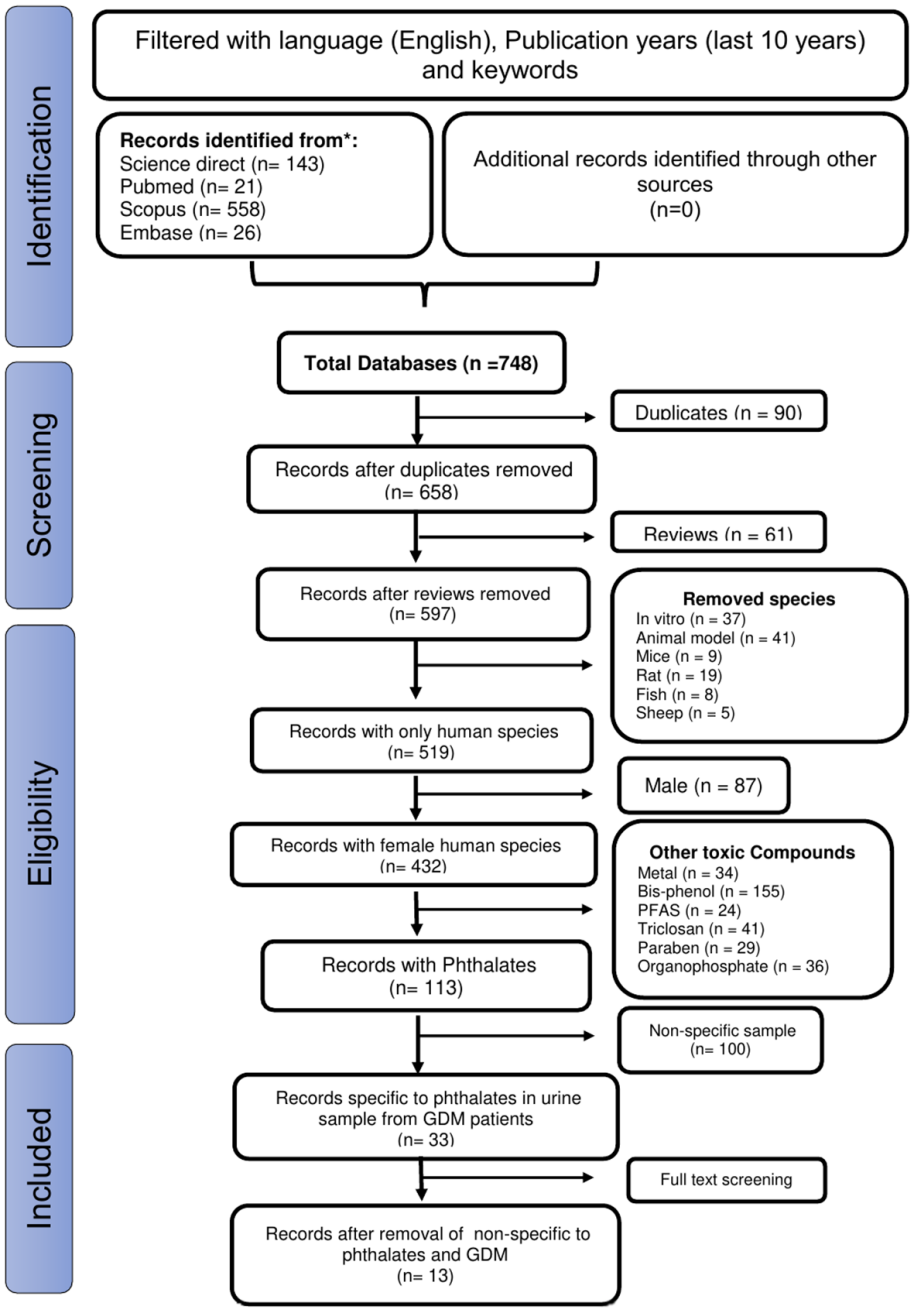
The PRISMA diagram shows the procedure used to select articles based on the predetermined inclusion and exclusion criteria.

**Figure 1** illustrates the study selection process. We retrieved 748 records and, after removing the duplicates and irrelevant articles, examined the titles and abstracts of the remaining 597 papers. Finally, after reviewing the full text of the remaining 33 papers, we identified 13 studies suitable for a systematic review (23–35). Age, Body Mass Index (BMI), parity, smoking status, and dietary patters were adjusted among all the pregnant women in the 13 studies.

### 3.2 Study characteristics

The 13 included studies, published between 2015 and 2025, focused specifically on human research. Research settings ranged from university laboratories to multicentered collaborations and private practices, with studies conducted in the United States of America (n=6), Canada (n=2), China (n=4), and Mexico (n=1). The procedure that was used to quantify the concentration of urinary phthalates was High Performance Liquid Chromatography (n=8), Gas Chromatography Mass Spectroscopy (n=2), Liquid Chromatography Mass Spectroscopy (n=1), Ultra-performance Liquid Chromatography-Mass Spectrometry (n=2) (**Table 1**).

**Table 1:** Overview of the studies included in the systematic review on phthalates and GDM.

Included study and year	Country of origin	Sample size (control/case)	Methods used for estimation	Quantity of phthalates	Creatinine adjustment	Pregnancy complication	Reference number
Shaffer et al., 2021	United States of America	705/150	HPLC	Trimester 1 - MIBP (2.4μg/L), MEP (3.8μg/L), MBP (2.4μg/L), MBZP (2.9μg/L), MCNP (2.8μg/L), MCOP (3.5μg/L), MCPP (3.6μg/L), MEHP (2.5μg/L), MEHHP (2.6μg/L), MEOHP (2.5μg/L), MECPP (2.4μg/L), DEHP (2.3 nmol/mL). Trimester 3 - MIBP (2.6μg/L), MEP (4.5μg/L), MBP (2.7μg/L), MBZP (3.2 μg/L), MCNP (2.7μg/L), MCOP (3.3μg/L), MCPP (3.7μg/L), MEHP (2.5μg/L), MEHHP (2.5μg/L), MEOHP (2.5μg/L), MECPP (2.3μg/L), DEHP (2.4 nmol/mL).	Yes	GDM, GWG	(23)
Todd et al., 2016	United States of America	251/47	HPLC	MEP (3.59µmol/L), MBP (1.27µmol/L), MIBP(1.79µmol/L), MBZP(1.13µmol/L), MCPP (0.98µmol/L), DEHP (0.48µmol/L)	Yes	GDM, GWG	(24)
Todd et al., 2022	United States of America	136/470	HPLC	MEP (58.79ng/mL), MBP (11.6ng/mL), MHBP (1.28ng/mL), MIBP (6.1ng/mL), MHIBP (2.46ng/mL), MBZP (4.26ng/mL), MCPP (2.31ng/mL), MEHP (3.79ng/mL), MEHHP (13.45ng/mL), MEOHP (9.34ng/mL), MECPP (21.74ng/mL), MEHHTP (3.06ng/mL), MECPTP (3.81ng/mL), MNP(1.54ng/mL), MONP (5.07ng/mL), MCOP (2.4ng/mL), MHiNCH (0.24ng/mL), MCOCH (0.25ng/mL), DEHP (0.17ng/mL).	Yes	GDM	(25)
Lang et al., 2024	China	100/65	GC-MS	Case - BBP(25.67ng/mL), DBP(80.71ng/mL), DEHP (15.92ng/mL), DEP (3.82ng/mL), DMP (3.11ng/mL). Control - BBP(24.86ng/mL), DBP(50.4ng/mL), DEHP (9.02ng/mL), DEP (1.99ng/mL), DMP (1.76ng/mL).	Yes	GDM	(26)
Soomro et al., 2024	Canada	405/15	HPLC	MMP(0.10µg/L), MEP (0.10 µg/L), MBP (0.10 µg/L), MIBP (0.10 µg/L), MECCP (0.10 µg/L), MEOHP (0.10 µg/L), MEHP (0.10 µg/L), MBZP (0.10 µg/L), MCOP (0.10 µg/L), MNP (0.10 µg/L), MCNP (0.10 µg/L), MCHP (0.10 µg/L), MOP (0.10 µg/L).	Yes	GDM	(27)
Chen et al., 2022	China	338/338	GC-MS	MMP (0.038μg/L), MEP (0.029μg/L), MIBP (0.002μg/L), MBP (0.002μg/L), MEHP (0.033μg/L), MOP (0.063μg/L), MBZP (0.041μg/L), MEOHP (1.07μg/L), MEHHP (0.010μg/L), MECPP (23.4μg/L)	Yes	GDM	(35)
Zukin et al., 2021	United States of America	99/316	LC-MS	MEP (184.6ng/ml), MBP (22.9ng/ml), MIBP (2.7ng/ml), MBZP (7.2ng/ml), DEHP (0.2ng/ml), MCPP (1.7nmol/ml), MCOP (2.9ng/ml), MCNP (1.8ng/ml)	Yes	GDM, GWG	(28)
Shapiro et al., 2015	Canada	1167/48	UPLC-MS	Case - MEP (34.5 μg/L), MBP (12.3μg/L), MBZP (6.3μg/L), MCPP (0.8μg/L), MEHP (2.7μg/L), MEHHP (11.4μg/L), MEOHP (7.8μg/L). Control - MEP (38.8 μg/L), MBP (13.3μg/L), MBZP (5.8μg/L), MCPP (1.0μg/L), MEHP (2.6μg/L), MEHHP (10.6μg/L), MEOHP (7.4μg/L).	Yes	GDM	(29)
Gao et al., 2021	China	2489/428	HPLC	DMP (0.93μg/mL), DEP (1.17μg/mL), DBP (1.05μg/mL), BBZP (0.94μg/mL), DEHP (0.94μg/mL)	Yes	GDM, GWG, HDP	(30)
Robledo et al., 2015	United States of America	57/15	HPLC	MBP (30.38μg/l), MIBP (11.22μg/l), MEHP (3.24μg/l), MEHHP (19.88μg/l), MEOHP (13.97μg/l), MECPP (33.28μg/l), MEP (216.42μg/l), MBZP (18.23μg/l), DEHP (188.07μg/l), DBP (63.53μg/l).	Yes	GDM, Obesity	(31)
Ibarra et al., 2019	Mexico	22/18	UPLC-MS	MBZP (1.71µg/l), MBP (93.15µg/l), MBIP (9.89µg/l), MEHP (11732µg/l)	Yes	GDM, Obesity, C-section	(32)
Liang et al, 2022	China	100/100	HPLC	MMP (7.58 μg/L), MEP (9.53 μg/L), MCHP (0.33 μg/L), MOP (1.1 μg/L), MINP (0.39 μg/L), MIBP (12.47 μg/L), MBP (107.81 μg/L), MBZP (0.51 μg/L), MEHP (3.26 μg/L), MEOHP (6.33 μg/L), MECPP (53.17 μg/L)	Yes	GDM	(33)
Todd et al, 2018	United States of America	235/10	HPLC	MEP (43.6ng/mL), MBP (10.9ng/mL), MIBP (5.7ng/mL), MBZP (3.0ng/mL), MCPP (4.9ng/mL), MCOP (28.2ng/mL), MCNP (4.2ng/mL), DEHP (0.2nmol/mL)	Yes	GDM, Obesity	(34)

MIBP, Mono-Isobutyl Phthalate; MEP, Monoethyl Phthalate; MBP, Monobutyl phthalate; MBZP, Monobenzyl Phthalate; MCNP, Mono-Carboxynonyl Phthalate; MCOP, Mono-carboxy-isooctyl Phthalate; MCPP, Mono(3-carboxypropyl) Phthalate; MEHP, Monoethylhexyl Phthalate; MEHHP, Mono(2-ethyl-5-hydroxyhexyl) Phthalate; MEOHP, Mono-(2-ethyl-5-oxohexyl) Phthalate; MECPP, Mono(5-carboxy-2-ethylpentyl) Phthalate; DEHP, Di(2-ethylhexyl) Phthalate; MHIBP, Mono-hydroxyisobutyl Phthalate; DMP, Dimethyl Phthalate; DEP, Diethyl phthalate; DBP, Dibutyl Phthalate; BBZP, Butyl Benzyl Phthalate; MCHP, Monocyclohexyl Phthalate; MOP, Mono-n-octyl Phthalate; GDM, Gestational Diabetes Mellitus; GWG, Gestational Weight Gain; HDP, Hypertensive Disorders of Pregnancy; C- section, Caesarean Section; HPLC, High Performance Liquid Chromatography; GC-MS, Gas Chromatography Mass Spectroscopy; LC-MS, Liquid Chromatography Mass Spectroscopy; UPLC-MS, Ultra-performance Liquid Chromatography-Mass Spectrometry.

### 3.3 Association of urinary phthalates with GDM

All 13 included studies showed an increase in the concentration of urinary phthalate concentrations among pregnant women with GDM compared to those of pregnancies without GDM (**Figure 1**). Our findings from the included studies showed not only the presence of secondary phthalate metabolites like Mono-Isobutyl Phthalate (MIBP), Monoethyl Phthalate (MEP), Monobutyl Phthalate (MBP), Monobenzyl Phthalate (MBZP), Mono-Carboxynonyl Phthalate, Mono-carboxy-isooctyl Phthalate, Mono(3-carboxypropyl) Phthalate, Monoethylhexyl Phthalate, Mono(2-ethyl-5-hydroxyhexyl) Phthalate, Mono-(2-ethyl-5-oxohexyl) Phthalate, Mono(5-carboxy-2-ethylpentyl) Phthalate, Monocyclohexyl Phthalate, and Mono-n-octyl Phthalate the urine sample of GDM patients but also showed a significant increase in the levels of primary phthalate metabolites like Di(2-ethylhexyl) Phthalate (DEHP), Dimethyl Phthalate, Diethyl phthalate, and Dibutyl Phthalate in the urine sample of GDM patients (**Table 1**) (**Supplementary Table S2**). The values of the most common phthalates among the 13 studies were converted to μg/L, and log10 scale values are also provided in the **Supplementary Table S2** to maintain homogeneity among the concentrations in these 13 studies.

Most of the studies inferred not only a significant rise in the concentrations of the secondary metabolites MEP, MBP, MIBP, and MBZP but also the primary metabolite DEHP in the urine samples of GDM pregnancies.

**Figure 2:**
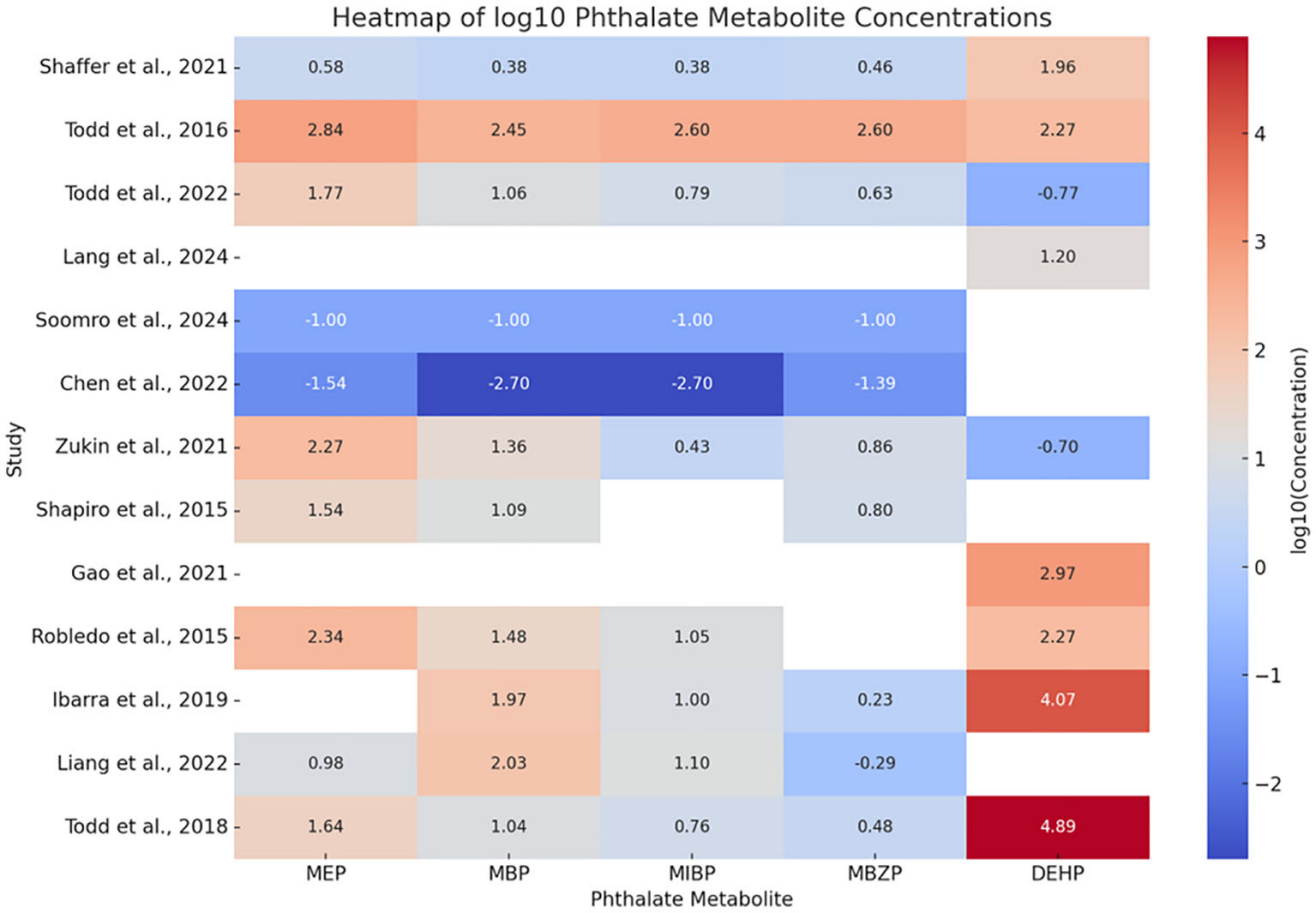
Comparison of the concentration of common phthalates between all 13 studies using a heat map that the authors have analyzed. The values from all 13 selected studies have been converted to µg/L to maintain homogeneity of values, followed by conversion to a log10 scale to eliminate existing extreme differences in the values. The red color in the log10 scale represents higher concentrations, while the blue color represents lower concentrations.

The comparison in the concentration of the phthalates that were most common among the 13 studies is shown using a heat map of the log10 phthalate concentration values in μg/L (**Figure 2**). 6 studies showed an elevated level of MEP (24, 25, 28, 29, 31, 34), 6 studies showed an elevated concentration of MBP (25, 28, 31–34), 2 studies showed an elevated concentration of MIBP (25, 34), 2 studies showed an elevated concentration of MBZP (25, 34), and 5 studies showed an elevated concentration of DEHP (23, 25, 30–32).

### 3.4 Pregnancy outcomes

Pregnancy outcomes following the diagnosis of the pregnant women from all included thirteen studies were broadly divided into primary and secondary outcomes.

The primary outcome of the study refers to the most common metabolic dysfunction affecting both the mother and the fetus in the studies included in the systematic review for data analysis. The primary pregnancy outcome that was seen commonly among the study participants recruited in the included studies is impaired glucose tolerance due to GDM (23–35). These studies show that women have greater fasting plasma glucose and post-prandial glucose concentrations than normal subjects because of higher amounts of systemic insulin resistance.

The secondary outcomes analyzed as part of this study focused on additional severe maternal and fetal outcomes of pregnancy that help to interpret the results of the primary outcome of GDM. The secondary outcomes included obesity, gestational weight gain, and hypertensive disorders of pregnancy (30, 32).

### 3.5 Risk of bias analysis

**Figure 3:**
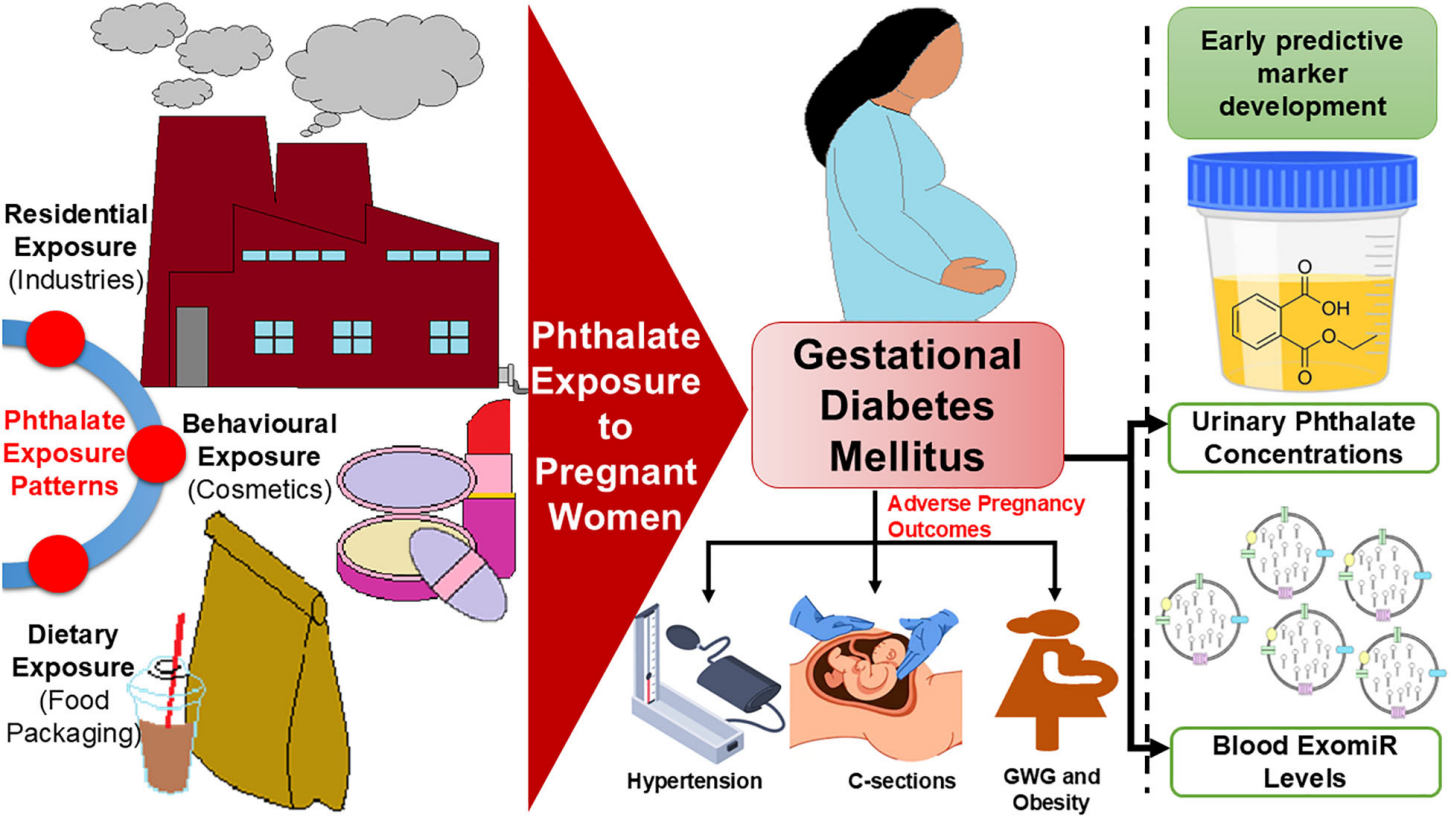
Analysis of risk of bias using the New Castle Ottawa Scale selection tool.

The current systematic review included 13 cohort studies, initially assessed for methodological quality using the Newcastle-Ottawa Quality Assessment Scale (NOS), which scores studies across three domains: selection, comparability, and outcome. Based on the NOS scores, further outcome-specific evaluations were carried out using the Risk of Bias 2 (RoB 2) tool. Risk of bias judgments were presented in tabular form (**Supplementary Table S3**) and visualized through traffic light plots to reflect the level of bias across studies. These assessments informed the interpretation of findings and the overall grading of evidence quality, identifying one study (33) as having a high risk of bias (**Figure 3**).

### 3.6 Additional findings

Other EDCs were also found to be significantly associated with GDM. Many studies also suggested associating urinary heavy metals levels like arsenic, lead, cadmium, manganese, mercury, antimony, copper, magnesium, molybdenum, selenium and zinc in GDM patients (27). The elevated levels of flame retardants like tris (2-butoxyethyl) phosphate, tributyl phosphate, tris (2-chloroethyl) phosphate, tris (1,3-dichloro-2-propyl) phosphate, tri-ortho-cresyl phosphate, and triphenyl phosphate were also seen to be associated with GDM (26). Studies also showed a significant increase in urinary bisphenols and perfluoroalkyl acid levels in GDM patients (27, 29, 32). A study also found that di(isononyl) cyclohexane 1,2-dicarboxylate was significantly associated (24).

In a study by Ibarra et al., they showed that elevated levels of phthalates are related to the overexpression of micro ribonucleic acids (miRs) in the serum samples of the patients, including miR-9-5p, miR-16-5p, miR-29-3p, and miR-330-3p (32).

## 4 Discussion

The studies included in this review highlight clear differences in both the types and concentrations of phthalate metabolites detected among pregnant women with and without GDM. This study is an alternative perspective on the impact of phthalates in gestational diabetes mellitus (GDM), emphasizing their role as endocrine-disrupting chemicals (EDCs) that interfere with metabolic and hormonal regulation during pregnancy (36). EDCs may interact differently with nuclear hormone receptors (e.g. PPARγ and estrogen receptors) in GDMA2 compared to GDMA1 due to hormonal imbalances and altered adipokine profiles. GDMA2 individuals tend to have greater degrees of insulin resistance and dysregulated glucose metabolism. EDCs including phthalates, BPA, and parabens have been linked to impaired β-cell function, disruption of insulin signaling, and increased oxidative stress (37). These effects may be more prominent in GDMA2, exacerbating the already existing metabolic abnormalities and potentially resulting to poor maternal and neonatal outcomes.

### 4.1 Effect of phthalates on metabolic pathways

Phthalates can bind to nuclear receptors such as peroxisome proliferator-activated receptors (PPARs), particularly PPARγ, disrupting adipogenesis, lipid metabolism, and insulin sensitivity, key pathways involved in the pathophysiology of GDM (36). This disruption may impair glucose uptake and exacerbate insulin resistance, especially during the second and third trimesters when insulin resistance naturally increases (36).

Moreover, the placenta is a critical mediator in fetal-maternal metabolic exchange and is highly sensitive to environmental toxicants (38). Studies suggest that phthalate exposure can lead to placental inflammation, oxidative stress, and changes in gene expression, all of which are linked to impaired glucose metabolism (38, 39). Epigenetic alterations, such as changes in DNA methylation or miRNA regulation, have also been implicated, potentially influencing both maternal glycemic control and fetal metabolic programming (40, 41). Furthermore, *in vivo* rat studies show gestational DEHP exposure impairs offspring glucose tolerance, while DBP worsens hyperglycemia and glucose handling (17). DBP also disrupts FOXM1, reduces β-cell viability, and impairs STAT1 signaling *in vitro (* 17).

Another significant consideration is that phthalate exposure is often not uniform across populations. Social and environmental determinants, including dietary habits (e.g., processed food consumption), use of personal care products, and occupational exposures, disproportionately affect certain groups, contributing to environmental health disparities (42).

### 4.2 Effect of phthalates on miRNA expression levels

The current systematic review aimed to assess the impact of phthalate exposure on the development of GDM. Evidence from epidemiological studies and mechanistic research indicates a positive association between phthalate exposure during pregnancy and impaired glucose regulation, increasing the risk of GDM. Numerous studies demonstrate that elevated urinary phthalate metabolites correlate with abnormal glucose metabolism in pregnant women (23). Higher phthalate exposure has also been linked with a greater incidence of GDM (25, 33, 35). Data from prospective cohorts further confirm these associations and highlight that trimester-specific exposure patterns may modulate the degree of risk (27, 29). Longitudinal evidence supports a link between phthalate exposure and disturbances in glucose levels and weight gain during pregnancy (30, 31). These associations are particularly evident in high-risk populations, emphasizing the disproportionate exposure burden among certain demographic groups (28). Epigenetic studies also suggest a mechanistic role through altered miRNA expression in pregnant women with GDM (32). Specifically, significant changes in miRNA patterns have been observed in GDM-affected pregnancies with high phthalate exposure, implicating pathways related to glucose metabolism and insulin signaling (17, 32). These findings support the integration of environmental exposure assessments into prenatal care protocols. Regulatory attention is warranted to limit phthalate exposure, particularly among reproductive-age women, to reduce GDM prevalence (1). Although this review synthesizes evidence from both human and experimental models, heterogeneity in study designs, exposure timing, phthalate types measured, and diagnostic criteria for GDM complicate direct comparisons across studies. Additional confounding factors may influence the observed associations, including diet, body mass index, and socioeconomic status. Moreover, many mechanistic insights are derived from animal models, which may not fully represent human metabolic responses (25, 26, 34). The impact of phthalates on GDM extends beyond direct metabolic disruption and involves complex interactions with hormonal pathways, placental function, and social determinants of health. Understanding these multidimensional effects is crucial for developing effective interventions and regulatory policies that protect maternal and fetal health.

### 4.3 Regional difference in phthalate profiles

The studies included in this review highlight clear regional differences in phthalate levels, with most studies originating from HICs such as the United States and Canada. In contrast, others were conducted in MICs regions like China and Mexico. Phthalate profiles and concentration levels varied remarkably by geographical regions. Possible reasons for wide variations could include differences in industrial use, consumer product regulations, and lifestyle factors such as food packaging, usage patterns of personal care products, and housing and living conditions. Additionally, the usage of different detection methods across studies resulted in methodological diversity. These varying regional trends in phthalate exposure underscore the importance of interpreting associations with GDM within local environmental and regulatory contexts, with a focus on regional variability in exposure sources when developing preventive strategies.

### 4.4 Co-exposure to other endocrine disruptors

Co-exposure to multiple environmental toxicants such as bisphenols, heavy metals, perfluoroalkyl acids, and flame retardants poses complex health risks due to potential additive or synergistic effects. Bisphenols disrupt endocrine pathways, while heavy metals like lead, mercury, and cadmium impair neurodevelopment and metabolic health (17, 43). PFAs are highly persistent, bioaccumulative, and linked to immune, hepatic, and reproductive dysfunctions (17, 44). Flame retardants, particularly polybrominated diphenyl ethers (PBDEs), interfere with thyroid regulation and neurodevelopment. Emerging evidence suggests that combined exposure may exacerbate oxidative stress, metabolic disorders, and developmental toxicity beyond individual chemical effects (17, 45). Hence, co-exposure assessment is vital for realistic risk evaluation in environmental health.

## 5 Conclusion

GDM, a transient hyperglycemic stage with higher than usual plasma glucose levels during pregnancy, has been linked to pregnancy issues such as hypertension, macrosomia, preterm delivery, preeclampsia, and stillbirths (46). GDM prevalence has grown dramatically, with reported frequencies ranging from 15% to 25% (47). Some data suggest that environmental contaminants may impair glucose homeostasis and glucose tolerance in healthy women. The risk of GDM and maternal exposure to phthalates were systematically reviewed in this study. This study’s findings revealed a link between phthalate exposure during pregnancy and the likelihood of GDM.

**Figure 4:**
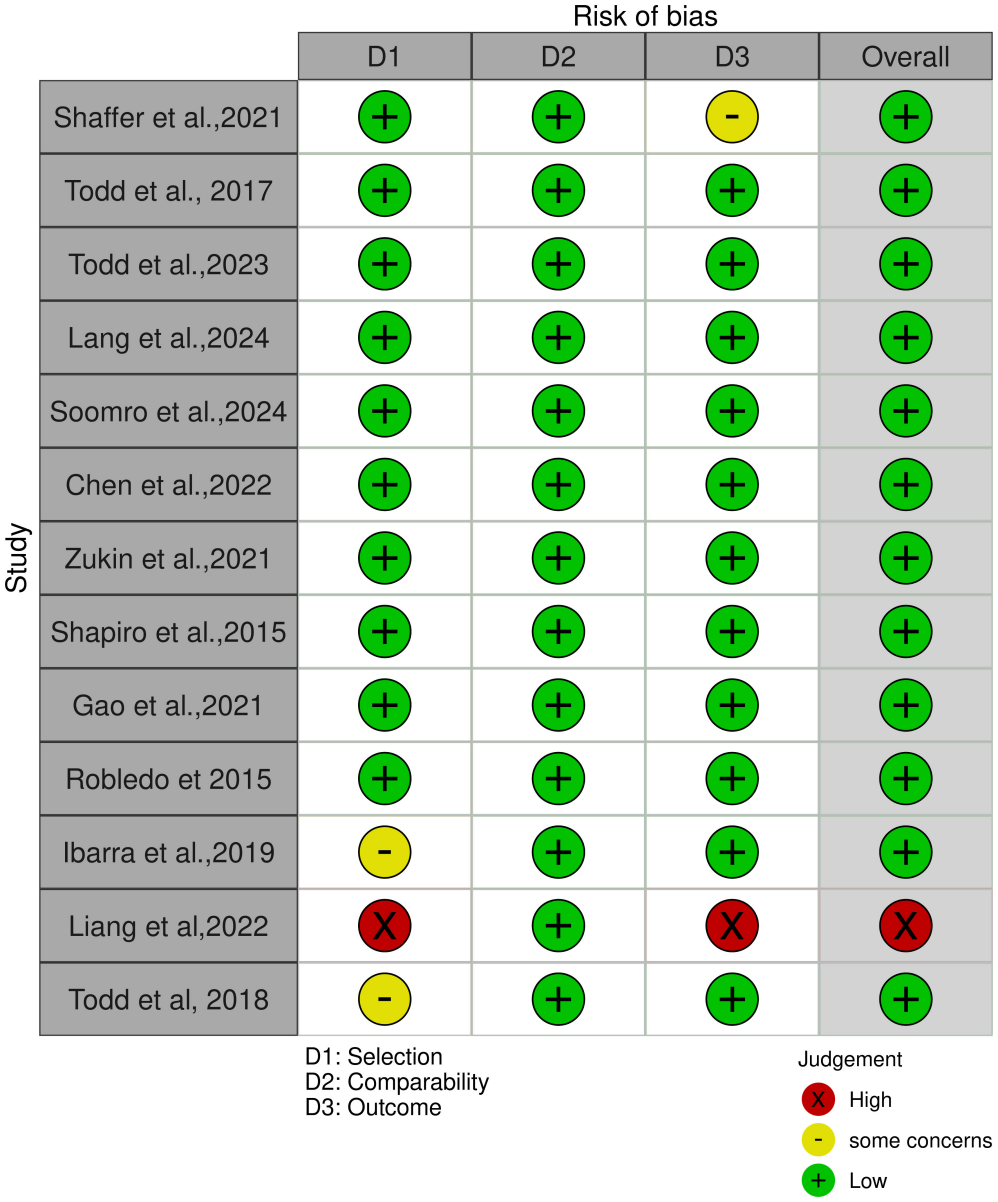
Depicts the exposure pattern of pregnant women to phthalates resulting in GDM and associated complications.

Our systematic review details the associations between phthalates and GDM pathophysiology, suggesting that urinary phthalates may serve as a predictor of GDM and associated complications. Phthalates significantly contribute to the development of insulin resistance, which often results in impaired glucose tolerance. These findings further underscore the importance of these plasticizers in the incidence of GDM. Additionally, phthalates are linked to pre-pregnancy BMI, which increases the likelihood of adverse pregnancy outcomes, such as HDP, GWG, and cesarean sections. Our findings in GDM patients align with previous research on phthalates concerning adverse maternal complications like HDP and gestational anemia (48), as well as neonatal complications, including various cardiovascular and neurological anomalies (13, 48–50). Furthermore, utilizing EDC-associated ExomiRs from patients’ blood can facilitate the early detection of GDM (13), enabling patients to be triaged based on escalating risk factors of the clinicopathologic illness (**Figure 4**). Single-point measurements of short-half-life phthalates like MEP and MBP may lack reliability as early GDM markers due to rapid metabolism and high intra-individual variability. While some studies link phthalate exposure to insulin resistance, longitudinal or repeated measurements are likely needed for robust prediction (51).

However, due to the scarcity of publications on this subject, it appears that the true impact of phthalate exposure remains unknown. As a result, more well-designed studies with a bigger sample size and longitudinal design are strongly suggested so that urinary phthalate levels can be strongly used as a non-invasive early predictive tool for GDM.

## 6 Limitations of the study

Some of the constraints encountered during this investigation are described below. First, the research design criteria may have resulted in a bias (selection bias) in the included studies. Future studies can circumvent this limitation by implementing and reporting on randomization, blinding, and the *a priori* technique. Second, only a few studies have been selected for subgroup analysis, which may limit the ability to detect the influence of xenobiotics, such as phthalates, on GDM. Moreover, the GDM patients included in the research population in all 13 investigations were not separated between GDMA1 and GDMA2, indicating a possible mechanistic difference. Furthermore, the authors didn’t separate the research population into early and late GDM. Patients who acquire GDM early in their pregnancy have a higher chance of developing gestational anemia. Finally, any dietary and lifestyle changes offered to patients during pregnancy may have a major impact on the concentration of urine phthalates, which must not be ruled out. Despite the variation across trials, similar effects of phthalates on individuals with GDM were discovered, justifying clinical translation efforts.

## Data Availability

The raw data supporting the conclusions of this article will be made available by the authors, without undue reservation.
